# Adsorption Isotherm Analysis for Hybrid Molecularly Imprinted Polymeric Gold-Decorated Nanoparticles Suitable for Reliable Quantification of Gluconic Acid in Wine

**DOI:** 10.3390/nano15030211

**Published:** 2025-01-28

**Authors:** Nelson Arturo Manrique Rodriguez, Marco Costa, Sabrina Di Masi, Christopher Zaleski, Alvaro García-Cruz, Giuseppe Mele, Vito Michele Paradiso, Sergey Piletsky, Cosimino Malitesta, Giuseppe Egidio De Benedetto

**Affiliations:** 1 Laboratory of Analytical Chemistry, Department of Biological and Environmental Sciences and Technologies, University of Salento, Via per Monteroni, 73100 Lecce, Italy; nelsonarturo.manriquerodriguez@studenti.unisalento.it (N.A.M.R.); cosimino.malitesta@unisalento.it (C.M.); 2 Department of Chemistry, University of Leicester, University Rd., Leicester LE1 7RH, UK; cz155@leicester.ac.uk (C.Z.); agc14@leicester.ac.uk (A.G.-C.); sp523@leicester.ac.uk (S.P.); 3 Department of Engineering of Innovation, University of Salento, via per Arnesano km 1, 73100 Lecce, Italy; giuseppe.mele@unisalento.it; 4 Laboratorio di Microbiologia Agraria e Tecnologie Alimentari, Dipartimento di Scienze e Tecnologie Biologiche ed Ambientali, Università del Salento, Via per Monteroni, 73100 Lecce, Italy; vito.paradiso@unisalento.it; 5 Laboratory of Analytical and Isotopic Mass Spectrometry, Department of Cultural Heritage, University of Salento, 73100 Lecce, Italy; giuseppe.debenedetto@unisalento.it

**Keywords:** molecularly imprinted polymer, liquid polymerisation, in situ gold nanoparticles, gluconic acid, amperometric sensor, kinetics investigation

## Abstract

A class of hybrid molecularly imprinted polymeric nanoparticles (nanoMIPs) comprising the in situ formation of gold nanoparticles (AuNPs) immobilised in a molecularly imprinted D-gluconate polymer has been designed with the objective of attempting the electrochemical quantification of gluconic acid (GA) in a wine setting. The imprinted polymers were synthesised in the presence of AuNP precursors in a pre-polymerisation mixture, which were confined to one another during the polymerisation of the chains. This allowed the formation of hybrid electroactive responsive imprinted nanoparticles (hybrid AuNPs@GA-nanoMIP), which exhibited enhanced electron conductivity. The morphological characterisation of the produced nanoMIPs revealed a fully decorated Au spherical surface of 200 nm in diameter. This resulted in a large active surface area distribution, as well a pronounced electrochemical peak response at the commercial screen-printed platinum electrode (SPPtE), accompanied by enhanced electron kinetics. The AuNPs@GA-nanoMIP sensor demonstrated the ability to detect a broad range of GA concentrations (0.025–5 mg/mL) with exceptional selectivity and reproducibility. The calibration curves were fitted with different isotherm models, such as the Langmuir, Freundlich and Langmuir–Freundlich functions. Moreover, the efficacy of the detection method was demonstrated by the recovery rates observed in real samples of Italian red wine. This research contributes to the development of a robust and reliable electrochemical sensor for the on-site determination of gluconic acid in food analysis.

## 1. Introduction

Gluconic acid (GA) plays a pivotal role in the field of oenology, serving as a critical indicator of grapevine health during the maturation process. In particular, it has been identified as a bioindicator of the disease caused by the fungus *Botrytis cinerea*, which affects the physio-chemical stability of wine [1,2,3]. This includes alterations to grape skin properties [4,5] and the polyphenol oxidative chain mechanism, as well as the induction of gluconic acid formation [6]. The presence of a high concentration of GA resulted in the production of wines with a botrytised character, displaying a distinctive luscious and apricot-like aroma with a subtle honey-like note. Therefore, in order to guarantee the quality of the product and to enhance the efficiency of the manufacturing process, it is essential to monitor the concentration of d-gluconate in these matrices. The d-gluconate content recommended by the International Organisation of Vine and Wine (OIV) is less than 0.3 mg/mL. Nevertheless, an early stage of fungal infection is indicated by values up to 1.0 mg/mL [7]. Analytical methods that employ high-performance liquid chromatography (HPLC) [8,9] coupled with an amperometric detector [10,11], or biosensor assays [7,12], have already been proposed for the quantification of organic acids in the wine industry. From these, biosensors were identified as significant competitors [13], but their reliance on expensive and delicate biological components renders them unsuitable for use in harsh industrial, field or environmental conditions [14].

Molecularly imprinted polymers (MIPs) have emerged as a significant technological advancement in the field of selective and sensitive analysis. An MIP is defined as a “plastic antibody” [15,16]. MIPs possess several advantages over their biological counterparts, including their low cost, ease of preparation, storage stability and applicability in various chemical media [17,18,19,20,21]. The application of MIPs in the quality monitoring of wine has been shown to result in the determination of numerous organic compounds in wine [22], including caffeic acid [23], ethanethiol [24] and quercetin [25]. Metal nanoparticles, such as silver or gold nanoparticles (AuNPs), offer improved electrical properties [26,27,28] when used in combination with electrochemical transductions [29]. AuNPs exhibit a large surface-to-volume ratio [30] and provide high electrocatalytic efficiency at electrode surfaces, making them suitable for electroanalytical applications [31,32]. The diversity in binding sites could create nonlinear sensor responses, affecting the analytical performance of the sensor, such as its concentration range and selectivity [33]. As a result, it is important to understand the polymer properties of the synthesised product [34]. A significant challenge for the advancement of sensors based on MIPs is to effectively convert the recognition event into a measurable analytical signal, with exceptional sensitivity properties.

In this study, we present a novel hybrid sensor that incorporates the in situ formation of AuNPs within the MIP structure. The hybrid composite comprises nano-sized particles (less than 250 nm) coated with AuNPs that are closely immobilised within the imprinted network of MIPs. The sensing mechanism of the Au-nanoMIPs is based on a N-isopropylacrylamide–acrylic acid (NIPAM-AAc) polymer network that undergoes a volume phase transition in response to D-gluconate at concentrations relevant to wine. The lin-log calibration plot of the nanoMIP-based GA sensor is linear from 0.05 to 2.5 mg/mL, showing an increase in sensor response with increasing concentration. The calculated LOD was 0.023 mg/mL, and the reproducibility, calculated as the average relative standard deviation (%RSD) of four different sensors tested, was 6.3%. In order to describe the binding and kinetics behaviour of the sensor, these data were fitted using various models. Langmuir, Freundlich and Langmuir–Freundlich isotherms were compared to describe the efficiency of imprinted polymers (MIPs) compared to non-imprinted ones (NIPs). The device has adequate selectivity, and its monitoring capability was evaluated in real media, specifically red wine, in which it proved to be a useful tool for testing the presence of gluconic acid in wine, encouraging future prospects for on-site use in wineries. To the best of our knowledge, an attempt has not yet been made to use MIPs for the analysis of gluconic acid in wine.

## 2. Materials and Methods

### 2.1. Chemicals

N-isoproprylacrylamide (NIPAm) MN ~40,000, N-t-butylacrylamide (TBAm) (~100%), N-methylenebisacrylamide (BIS-MBAA) (99%), Chloroauric acid trihydrate (HAuCl_4_) (≥99.9%), acrylic Acid (AA) (99%), 3-aminopropyl methacrylamide HCl (AMP HCl) (98%), D-gluconic acid sodium salt (≥99%), Acetylacetone (AAc), hydrogen peroxide (H_2_O_2_) (35%), N-hydroxysuccinimide 99% (NHS), -(3-dimethylaminopropyl) -3-ethylcarbodimide hydrochloride 98% (EDC) (99%) and (3-Aminopropyl) triethoxysilane (APTES) (99%) were purchased from Sigma-Aldrich (Dorset, UK). A phosphate-buffered saline tablet (5 mM, pH 7.4) was purchased from Fisher Bioreagents (Leicestershire, UK). All the solutions were freshly prepared using PBS buffer solution prepared in ultrapure water. The stock solution of a gluconic acid 0.1 M solution was prepared and diluted to the tested concentration in mg/L. All tests reported the use of D-gluconate in the form of a salt: because its pKa = 3.7, gluconic acid dissociates in water at pH 7, giving the gluconate anion. For the selectivity test, the following competitive interferents (commonly found in wine samples) were used: d(-)-fructose, malic acid and glucose (≥99.5%). For the real matrix test, Italian red wine was purchased from a local shop.

### 2.2. NanoMIP Molecular Modelling

To design the molecular modelling of D-gluconate imprinted nanoMIPs, Sybyl 7.3 software (Tripos Inc., St. Louis, MO, USA) was used. For the synthesis, NIPAm, AA, AMP HCL and BIS-MBAA as were chosen as functional monomers, TBAA as a cross-linker agent, hydrogen peroxide as an initiator, HAuCl_4_ as a Au nanoparticle precursor and AAc (2:1) as a reducing agent.

### 2.3. Synthesis of Hybrid AuNPs@GA-nanoMIPs and Control

In a 15 mL vial, 1 mL of aqueous solution of GA (5.0 mg, 1 mM) followed by the addition of NIPAM (390 μL, 21.2 mM), AA (50 μL, 2 mM), TBAA (300 μL, 14.5 mM, dissolved in ethanol), BIS-MBAA (250 μL, 2.4 mM), AMP HCl (120 μL, 2.1 mM), HAuCl_4_ (200 μL 0.08mM), H_2_O_2_ (300 μL, 651mM) and 500 μL of Acetylacetone/Acetone (2:1) were allowed to undergo a polymerisation reaction under N_2_ flow for 10 min. To remove gluconic acid and unreacted monomers, the hybrid dispersion (15 mL) was centrifugated using centrifuge filter tubes from Amicon^®^ Ultra (10 MWCO) at 3500 rpm for 20 min (×6), with the precipitated solution redispersed in 15 mL ultrapure water after each cycle (×6). At the conclusion of the centrifugation process, the collected 3 mL was concentrated to a volume of 1.5 mL by evaporation under inert gas (N_2_), thus producing the washed AuNPs@GA-nanoMIPs. The control (non-imprinted) was prepared in an identical manner but in the absence of a template.

### 2.4. Characterisation of Hybrid AuNPs@GA-nanoMIPs

The morphological and dimensional characterisations of AuNPs@GA-nanoMIPs and nanoNIPs were carried out by transmission electron microscopy (TEM) and dynamic light scattering (DLS), respectively. DLS measurements were performed using a Zetasizer Nano (Malvern Instruments Ltd., Malvern, UK). To acquire TEM images, a JEOL JEM-1400 TEM coupled with an accelerating voltage of 120 kV was utilised. Digital photos were collected using the EMSIS Xarosa 20MP digital camera and Radius software (https://www.radius.com/). A Scientific Ltd. 400 mesh AGS160 Agar carbon film grid was discharged with 10 µL of the material sonicated on it. In a Quorum GloQube system, grids were glow-discharged for 15 s at 20 milliamperes. After this, the sample was adsorbed and dried at room temperature for twenty-five minutes.

The Alpha II platinum ATR FTIR spectrometer (Bruker, Coventry, UK) was used for Fourier-transform infrared spectroscopy (FT-IR) analysis to characterise the nanoparticles before and after washing treatment. The electrochemical measurements were conducted using a portable potentiostat (PalmSens 4, PalmSens 4, PalmSens BV, Houten, The Netherlands), while data acquisition was performed with PSTrace5 software, v. 5.9 (PalmSens, PalmSens BV, The Netherlands). Additionally, nanoMIPs and the control were characterised by localised surface plasmon resonance (LSPR) technique measurements using NanoSPR 103 (NanoSPR, Chicago, IL, USA). Prior to the acquisition of the LSPR spectrum, dark and reference signals for background noise cancellation were measured using a glass slide as a reference.

### 2.5. Fabrication and Optimisation of Electrochemical Sensor

The functionalisation of the screen-printed electrode (SPPtE, Dropsens DRP-550 AT, Metrohm, Runcorn, UK) was studied to optimise the optimal coverage of the working surface. For this purpose, four different concentrations of APTES (0.5%, 1.0%, 2.5% and 5%) dissolved in ethanol were used for the functionalisation. Subsequently, electrochemical impedance spectroscopy (EIS) analyses were performed using [Fe(CN)_6_]^3−/4−^ as an electrochemical probe. The surface of the screen-printed platinum electrodes was subjected to chemical functionalisation with the optimal condition. To this end, 5 µL of a 3-aminopropyltriethoxysilane (APTES, 5% *v*/*v*) ethanolic solution, acting as the modifier, was drop-cast for 10 min. The procedure was repeated twice, after which the SPPtE was dried at 150 °C (1 h) in an oven. AuNPs@GA-nanoMIPs were immobilised onto the modified electrode with EDC as follows: First, two different solutions were prepared in PBS pH 7.4 5 mM for EDC (20 mg in 250 µL) and NHS (30 mg in 250 µL). Then, a solution comprising 80 μL of suspended AuNPs@GA-nanoMIPs, 10 μL of EDC and 10 μL of NHS (1:1.5) was prepared and drop-cast on the modified SPPtE for 40 min. Finally, the electrode surface was gently rinsed with MilliQ water to remove the unreacted chemicals and to ready it for use.

### 2.6. Electrochemical Sensing of D-Gluconate and Binding Isotherms

Cyclic voltammetry (CV) and differential pulse voltammetry (DPV) were employed to examine the electrochemical performance at each stage of electrode modification in 5mM PBS at pH 7.4. The potential range of CV was between −0.1 V and +0.8 V (vs. a pseudo-Ag|AgCl reference) at a scan rate of 50 mV/s. The parameters of the DPV measurements were as follows: the potential range was from −0.15 to +0.4 V (vs. Ag|AgCl), the modulation amplitude was 200 mV, the modulation time was 20 ms, the step potential was 4.95 mV, and the scan rate was 0.05 mV s^−1^. AuNPs were synthesised by drop-casting as follows: 94.6 mL of MilliQ water and 400 µL of 0.125 mol L^−1^ HAuCl_4_ were mixed on the heater; then 5 mL of 10 mg/mL sodium citrate was added under stirring. As AuNPs formed, the colour of the solution changed from yellow to red. Finally, the AuNP suspension was concentrated by centrifugation, and DLS analysis was performed (Figure S4). The sensor calibration for GA was carried out by drop-casting 100 µL of GA solutions, ranging from 0.0025 to 5.0 mg/mL, on the electrode surface, which was initially wetted with 5 mM PBS pH 7.4 (blank). After 10 min of incubation, the sensor was gently washed with MilliQ water and the DP voltammograms were recorded: they exhibited a formal peak at 0.1 V vs. Ag|AgCl, and the net peak current (Δi), i.e., the GA peak current subtracted from the PBS blank signal, varied in accordance with concentration. The incubation time of 10 min was chosen arbitrarily, according to the conditions that make an acceptable, fast and reliable analysis time. The incubation time in adsorptive voltammetry [35] is an important factor in detection performance as it affects the interaction between the analyte and the recognition sites present on the imprinted nanoparticles. The same procedure was utilised to obtain the calibration curve of the control (AuNPs@nanoNIP). The LOD was calculated as LOD = 3 × Sy/m, where *Sy* is the standard deviation of the intercept and *m* is the slope of the regression plot. The calibration curves were fitted using different approaches [36], specifically, with the Langmuir isotherm (LI), the Freundlich isotherm (FI), and the Langmuir–Freundlich (L-FI) models, whose equations are shown in Table 1, using OriginPro Lab 2016.

The Langmuir isotherm (1) is used for adsorption studies and assumes that all binding sites on the surface are equal, demonstrating the relationship between the amount of bound analyte (*q_e_*) and the free analyte in the system (*C_eq_*) at equilibrium, while $q_{m}$ describes the maximum adsorption capacity and *K_L_* is the Langmuir affinity constant. The Freundlich isotherm model (2) assumes that sorption occurs on a heterogeneous surface and that sorbed molecules interact in a reversible and non-ideal sorption process [37]. In the equation, *K_F_* is a measure of the adsorption capacity of the polymer for the target analyte, while *n* (heterogeneity exponent) describes the heterogeneity of the adsorption sites on the polymer surface. If the FI model is valid, an isotherm plotted in log-log format (log *C_e_* vs. log *q_e_*) is a straight line. Finally, *n*, a measure of the heterogeneity of a system, is obtained from the slope of the straight line of fit. To this end, $q_{e}$ was calculated as follows: $q_{e}=K_{F}\;C_{e}^{1/n}$. The calibration curve was also fitted with the Langmuir–Freundlich model (3), which has the advantages of being able to be used without making assumptions about heterogeneity and of overcoming the practical limitations in measuring the binding isotherm [38]. The maximum adsorption capacity (*Q_m_*), the affinity constant (*K_d_*) and the binding heterogeneity (*n*) were obtained through this model.

### 2.7. Selectivity and Electrochemical Evaluation in a Real Sample

The nanoMIP-based sensor selectivity was tested against potential interferents with structures similar to the target analyte. Accordingly, three organic compounds, namely, glucose, fructose and malic acid, used as acidifiers to correct the acidity of must and wine [39], were selected, and their responses on the AuNPs@GA-nanoMIP sensor were evaluated. Furthermore, Italian red wine was used to assess the potential utility of the proposed sensor for the selective detection of d-gluconate in authentic samples. Prior to analysis, the wine sample was mixed with phosphate-buffered saline (PBS) at a ratio of 1:10 (PBS:sample), and recovery was calculated at two different GA concentrations.

## 3. Results and Discussion

### 3.1. Molecular Modelling Design of Hydrid AuNPs@GA-nanoMIPs

Using the method provided by Sybyl, a database of common functional monomers for MIP synthesis was evaluated to formulate the recognition cavities of polymers specific to D-gluconate (C_6_H_11_O_7_) [40]. This enables the identification of suitable computational models for the rapid determination of appropriate monomers [41,42]. Strong interactions can be determined computationally by the algorithm, which uses atom-like particles, charge distribution and classical potentials, and selects a polymer for interaction by screening the library of virtual monomers [43]. It then places the functional monomers around the target and calculates the potential energy, electronegativity and interactions. Favourable interactions are evaluated in the form of bond values, with negative energies indicating suitable candidates. The Powell method combined with Tripos and Gasteiger–Huckel force fields and charges with a minimum energy of 0.001 kcal mol^−1^ was used to optimise the geometry of the monomers. Molecular modelling showed that the length of the d-gluconate bonds was shortened after complexation with the monomers. All screened monomers (Figures S1 and S2) bind strongly to D-gluconate (SM1), but AA and MBA are the best functional monomers in terms of binding to D-gluconate, with a binding energy of −16.125 and −25.472 kcal/mol^−1^, respectively (Figure S3). Like in biological systems, affinities greater than −15 kcal/mol^−1^ are so tight that a target will most likely never dissociate before the receptor is degraded [44]. The capacity to rapidly and effectively assess a large number of potential MIP designs and choose the most promising ones for synthesis is one of the key benefits of designing MIPs using computer modelling. This strategy can boost the probability of success and drastically cut down on the time and resources needed to create new imprinted nanoparticles [45]. Furthermore, by reducing the amount of reagents that need to be tested for the polymerisation process, this theoretical strategy also helps to create a molecular approach that is more ecologically friendly.

### 3.2. Reaction Mechanism and Characterisation of AuNPs@GA-nanoMIPs

**Scheme 1:**
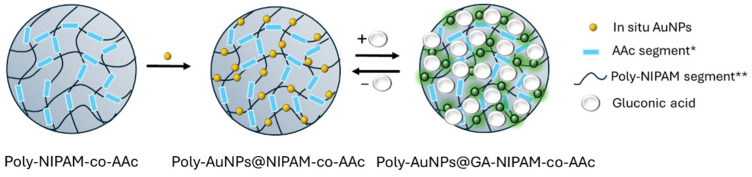
A possible reaction scheme during the formation of AuNPs@GA-nanoMIPs is shown. The sensing mechanism may involve an ON–OFF response triggered by the swelling behaviour of NIPAM upon molecule recognition (* AAc: acrylic acid; ** Poly-NIPAM: poly(N-isopropilacrilammide)).

Scheme 1 depicts a potential reaction pathway during the formation of AuNPs@GA-nanoMIPs. Throughout the polymerisation process, the poly-NIPAM-co-AAc polymer provides multiple amine, amidogen and carboxyl groups. The target molecule, GA (in its anionic form), contains hydroxyl and carboxyl groups, allowing the growing poly-NIPAM-co-AAc polymer to bind with GA via multiple hydrogen bonds. The noble metal trapping during chain propagation is likely attributed to the formation of Au-NH_2_ bonds between the nitrogen atoms from NIPAm and MBA. This process results in electroactive nanoparticles that are self-regulated in the presence or absence of the target analyte due to the fascinating properties of swelling typically observed in NIPAM-AA-based polymers [46].

**Figure 1:**
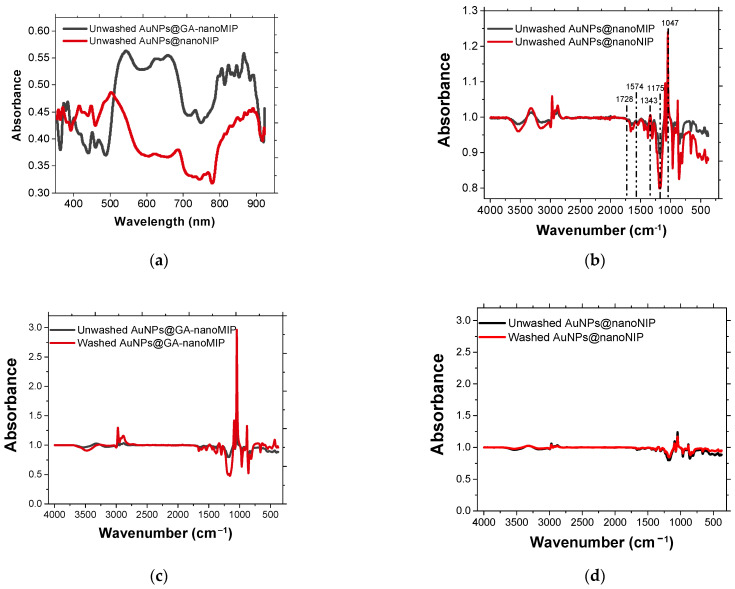
(**a**) LSPR absorption spectra recorded for unwashed AuNPs@GA-nanoMIP and AuNPs@nanoNIP. (**b**) ATR-FTIR recorded for (**b**) unwashed AuNPs@GA-nanoMIP compared to unwashed AuNPs@nanoNIP, (**c**) unwashed AuNPs@GA-nanoMIP compared to washed AuNPs@GA-nanoMIP and (**d**) unwashed AuNPs@nanoNIP compared to washed AuNPs@nanoNIP.

Localised surface plasmon resonance (LSPR) characterisation is proposed to demonstrate the plasmonic attribute of noble metals trapped on nanopolymers. As seen in Figure 1a, an LSPR peak of nanoMIP-capped AuNPs appeared at 550 nm, where the absorbance of Au can occur [47]. The LSPR peak of the control (unwashed nanoNIPs) resulted in a red-shift to 500 nm due to its different particle shape, charge and dispersion, because it was influenced by the presence of the analyte during the polymer growth of the imprinted nanoparticles, which modulates the polymer network in a different manner than that observed in the absence of the analyte [48,49]. Furthermore, peaks at the highest wavelengths are presumably caused by nanoparticle aggregation [50], where interparticle coupling leads to red-shifted plasmonic modes.

**Figure 2:**
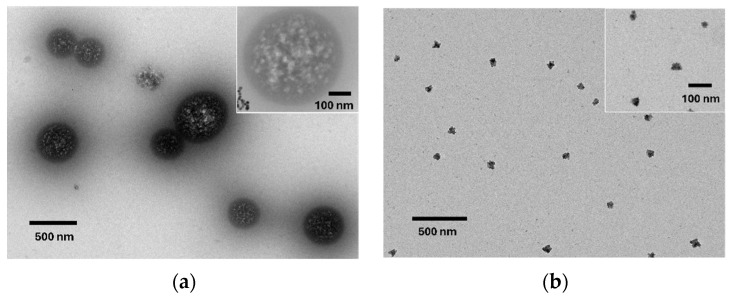
TEM image for (**a**) AuNPs@GA-nanoMIP and (**b**) AuNPs@nanoNIP after polymerisation reaction.

The ATR-FTIR spectra of unwashed nanoMIPs compared to the control are shown in Figure 1b. The bands located in the region between 3700 and 2700 cm^−1^ are present for both nanomaterials, indicating characteristic peaks of acrylate-based polymers. For the polymer control, typical bands of poly-NIPAM-co-AAc are visible: the bands at 1175 and 1047 cm^−1^ are attributed to C-O, C-O-C and C-OH stretching [51]. The characteristic -C=O stretching vibration band for amine II [52] is visible at 1539 cm^−1^. Finally, the poly-NIPAM structure is also confirmed by the peak at 1343 cm^−1^, which is attributable to the branch -CH(CH_3_)_2_ group. In the case of nanoMIPs, a low-intensity band at 1728 cm^−1^ is visible, which can be attributed to the carboxyl group of AAc [53]. Thus, it was confirmed that the imprinting process was successful. In addition, the intensity of the peak at 1721 cm^−1^ related to the AAc structure increases compared to that of the nanoNIP, suggesting that the contribution is due to the binding with the template. In the case of nanoMIPs, the intensity bands in the region of 1300–1000 cm^−1^ were modelled, indicating structural changes in the polymer, which ultimately led to target removal (Figure 1c). In the case of the AuNPs@nanoNIP, in the spectra obtained before and after the treatment, as expected, no visible changes occurred (Figure 1d). The DLS results estimated a particle size diameter between 100 and 300 nm, with a PDI at 0.265 (Figure S4), indicating that the particles are homogeneous and uniformly distributed. The TEM image (Figure 2) for the nanoMIPs shows well-dispersed particle morphologies, with a similar spherical shape about 250 nm in diameter, which results in good agreement with the first estimation from the DLS results.

However, the TEM images obtained for the AuNPs@GA-nanoMIP show a 2D representation of an MIP particle with a core of AuNPs more concentrated in the centre. The acquired images are in agreement with those of other gold-coated nanoparticles synthesised in previous works present in the literature [54,55]. This interesting result confirms the trapping of AuNPs, formed along the polymerisation chain growth. Moreover, the imprinted particles were much larger than those obtained for NIPs, indicating a different rearrangement of the polymer structure during the polymerisation reaction. At the end of polymerisation, the nanoNIPs are likely to be more heterogeneously dispersed with a wedge structure. These results suggest that the nano-sized particles prepared are ideal for attachment to the surface of screen-printed electrodes, which can guarantee a high surface area and more control over their distribution on the electrode surface.

### 3.3. Fabrication of Sensor and Electrochemical Characterisation

A bare SPPtE was chosen for the preparation of the electrochemical sensor. Prior to modification, the electrode was rinsed with ethanol and dried. Subsequently, 5 µL of an ethanolic solution of APTES was drop-cast for 10 min to obtain APTES/SPPtE via the covalent bond between the silane and the Pt material [56]. A concentration of 5% *v*/*v* 3-aminopropyltriethoxysilane (APTES) in ethanol demonstrated effectiveness for the functionalisation of the screen-printed electrode when considering the R_ct_ obtained by EIS measurements (Figure S5). APTES/SPPtEs have primary -NH2 groups available for further chemistry. Consequently, the prepared AuNPs@GA-nanoMIPs were attached to the electrode surface by the EDC/NHS coupling reaction [57,58], resulting in a stable amide bond.

**Figure 3:**
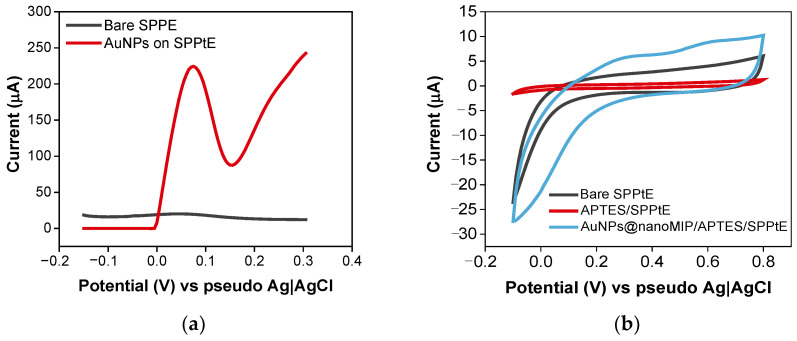
(**a**) DPV response at the bare electrode of the blank (PBS 5 mM, pH 7.4) and in the presence of AuNPs. (**b**) CV characterisation performed in PBS solution (5 mM pH 7.4) for (black line) the SPPtE, (red line) functionalised APTES/SPPtE and (blue line) AuNPs@GA-nanoMIP/APTES/SPPtE.

The electrochemical characterisation at each stage of modification was also explored to demonstrate the successful attachment of the recognition element. Preliminarily, the electrochemical activity of AuNPs at the bare surface of the SPPtE was investigated (Figure 3a) to ascertain its electrochemical readout signal.

The CV of the SPPtE in PBS at pH 7.4 did not show any significant electrochemical activity in the range from −0.2 V to 0.4 V vs. pseudo-Ag|AgCl, as expected. The addition of AuNPs produced a high-intensity DPV response peak at a potential of +0.07 V vs. pseudo-Ag|AgCl, which is attributed to the oxidation of gold at the electrode surface [59]. These results were confirmed by the CV characterisation obtained for the APTES/SPPtE and after AuNPs@GA-nanoMIP immobilisation. No evident electroactivity was recorded after APTES modification (red line) compared to the bare electrode. The integration of AuNPs@GA-nanoMIPs led to a significant increase in the current with respect to the APTES electrode, improving the electron transfer at the SPPtE surface. The observed CV results for the AuNPs@APTES/SPPtE represent the reversible reaction during the oxidation/reduction of Au observed at +0.07 V and −0.1 V vs. Ag|AgCl, respectively [60], making the nanoparticles electroactive. This means that the detection mechanisms at the cavities are strongly influenced by the electroactivity of Au.

### 3.4. Sensing Performance of AuNPs@GA-nanoMIP Device

**Scheme 2:**
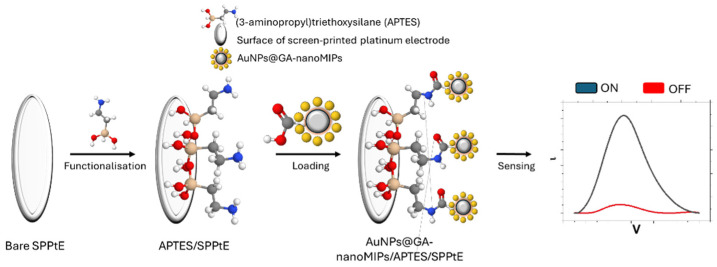
Sensor preparation based on hybrid AuNPs@GA-nanoMIPs and its sensing mechanism.

Scheme 2 illustrates the sensor preparation based on AuNPs@GA-nanoMIPs and the principle for the detection of GA.

Scheme 2 illustrates the sensing mechanism, showing how the analyte interacts with the imprinted molecule, leading to an oxidative current peak around 0.1 V vs. Ag/AgCl, which is linked to the presence of AuNPs in the polymer structure. At the recognition site, the interaction between imprinted molecules is readily converted into an electron flow pathway, with Au facilitating the signal conversion at the electrode surface, enabling the observation of the electrochemical signal (ON signal). Simultaneously, the signal diminishes in the absence of the template or in the presence of a known concentration of competing interferents (OFF signal). This allows the sensor to mimic enzymatic activity, with the recorded signal resulting solely from the direct recognition of the imprinted molecule, further enhanced by the presence of noble metal nanoparticles within the polymer network.

**Figure 4:**
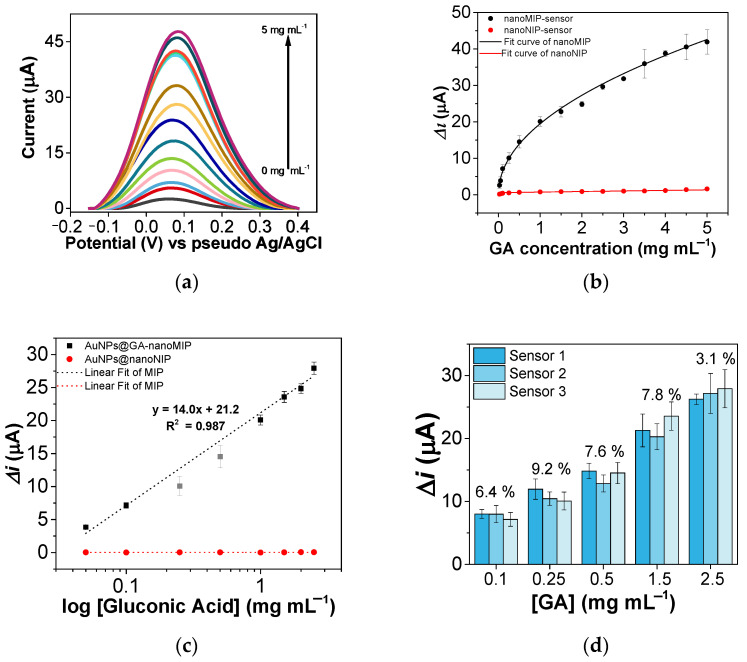
(**a**) DPV response of AuNPs@GA-nanoMIP sensor at increased concentration of GA (0.025 to 5 mg/mL) recorded in PBS buffer (5 mM, Ph = 7.4). (**b**) Calibration curves obtained for AuNPs@GA-nanoMIP and AuNPs@nanoNIP. (**c**) Working linear range of sensors at logarithmic concentration scale (from 0.05 mg/mL to 2.5 mg/mL). (**d**) Inter-sensor reproducibility of AuNPs@GA-nanoMIP sensors (n = 3) for different concentrations.

Figure 4a shows the affinity of the proposed sensor for GA. In fact, the peak currents at 0.07 V vs. Ag|AgCl increase with increasing concentrations of GA up to 5.0 mg/mL. As aforementioned, the presence of GA molecules in the MIP provides pathways for electron transfer, enabling redox conversion at the capped AuNPs [26], possibly through the template interaction within the recognition cavities by hydrogen bonding and shape complementarity. Furthermore, the presence of the tightly bound AuNP–polymer composite resulted in higher DPV current responses to nanoMIPs than the control. As a comparison, the DPV responses to GA of AuNPs@nanoNIP are shown in Figure S6. Furthermore, the calibration curve obtained for the imprinted nanoparticles via DPV measurements versus analyte concentration shows two linear regions (Figure S7). This behaviour can occur when a wide concentration range is tested and is typical for systems based on molecularly imprinted polymers, as reported in several previous publications [61,62,63,64].

The sensor responses were fitted with different isotherm model functions, and the obtained results are shown in Table 2. The parameters obtained from the LI, FI and L-FI models provide a comprehensive view of the adsorption properties of the synthesised polymers, both for the AuNPs@GA-nanoMIP and AuNPs@nanoNIP. In particular, according to the LI, the *Q_max_* value of 44.18 mg/g indicates the good maximum adsorption capacity of the AuNPs@GA-nanoMIPs compared to the AuNPs@nanoNIP, whose value is 12.34 mg/g. Furthermore, the imprinted polymer obtained a high *K_L_* value (1.03 L/mg), describing a strong affinity between the specific sites and the target analyte, against 0.05 L/mg of the non-imprinted polymer. According to the Freundlich isotherm, the *K_F_* of the imprinted nanoparticles is equal to 19.48 (mg/g) × (L/mg)^1/n^, compared to 0.65 (mg/g) × (L/mg)^1/*n*^ for the non-imprinted nanoparticles, confirming the significant adsorption capacity of the AuNPs@GA-nanoMIPs. The value of *n* (0.50) highlights a heterogeneity in the binding sites, consistent with the complex structure of the AuNPs@GA-nanoMIPs that presents, in addition to the nonspecific binding sites, cavities on the surface. Finally, the *Q_m_* obtained by the L–FI (139.18 mg/g) is higher than the *Qmax* obtained by the LI, indicating the possibility of adsorption on additional or less specific sites. The value of *K_d_* of the AuNPs@GA-nanoMIPs (0.05 M) compared to that of the AuNPs@nanoNIP (0.43 M) indicates the higher affinity of the AuNPs@GA-nanoMIPs towards the target, confirming at the same time the predominance of nonspecific interactions in the AuNPs@nanoNIP. Furthermore, the value of *n*, higher than that obtained in the previously mentioned fit, indicating a reduced heterogeneity compared to the results of the FI model, indicates that the L-FI represents a more realistic description of the system. The data described above indicate that the imprinted nanoparticles have better performance than the non-imprinted nanoparticles in terms of adsorption capacity (*Q_max_*, *Q_m_*) and affinity (*K_L_*, *K_F_*, *K_d_*). In the case of the FI, the isotherm was linearised, and the calibration curve was obtained (Figure S8). The obtained slope confirms the relative heterogeneity of the AuNPs@GA-nanoMIP surface compared to the AuNPs@nanoNIP [38].

The sensitivity of AuNPs@GA-nanoMIPs and AuNPs@nanoNIPs was calculated to be 14.0 ± 0.8 µA/decade and 9 × 10^−3^ ± 8 × 10^−3^ µA/decade, respectively (Figure 4c). The limit of detection (LOD) was 0.023 mg/mL. The average relative standard deviation (% RSD) of four different sensors was calculated to be 6.3% (Figure 4d), demonstrating acceptable inter-sensor reproducibility.

**Figure 5:**
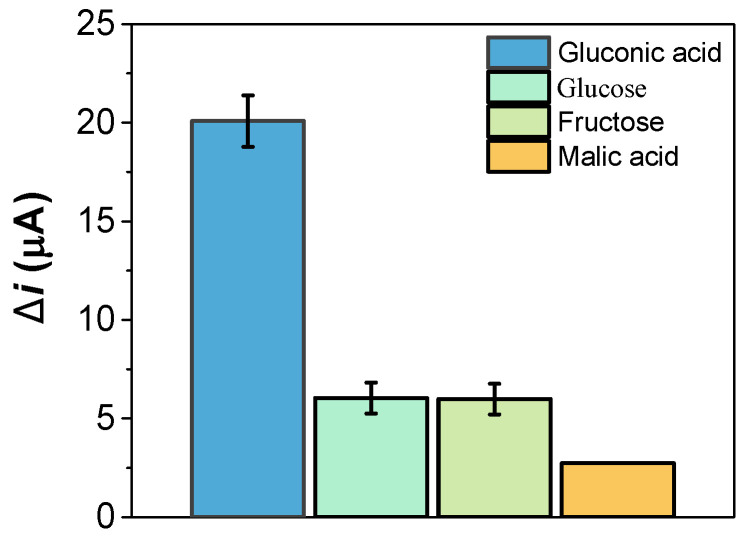
Selectivity study of AuNPs@GA-nanoMIPs towards gluconic acid and interferent compounds, such as malic acid, fructose and glucose, at 1 mg/mL.

The selectivity towards structural and competitive interferents of GA of the AuNPs@GA-nanoMIP sensor was investigated. The glucose molecule was chosen as a molecule of structural similarity, and fructose and malic acid were chosen as common organic compounds that are found in real wine samples. At a fixed concentration of 1 mg/mL, five different sensors were prepared and tested against these compounds. In general, the response of the AuNPs@GA-nanoMIP sensors to the studied interferents show negligible effects, as shown in Figure 5, where the responses at 1 mg/mL are represented. At lower concentrations, the responses of the nanoMIP-based sensor against the interferents are minimal (Figure S8).

At 1 mg/mL, the comparison of the DPV responses obtained with the target and the interferents confirms the applicability of the sensor for GA detection, as the response towards glucose and fructose was three times lower and that towards fructose was seven times lower. However, the overall responses can be considered reasonable due to the high concentration tested and given the similarity of the molecules. Recovery was measured at three different GA levels using *Primitivo* red wine samples as a matrix. As evidenced by the data in Table 3, the sensor also had good recovery at different levels in complex matrices such as red wine.

Finally, Table 4 compares our hybrid AuNPs@GA-nanoMIP sensor with some enzymatic sensors for GA detection.

In particular, the proposed AuNPs@GA-nanoMIP sensor has a low limit of detection (LOD) compared to others. However, its main advantages are related to its simple synthesis method, relatively low production cost, intrinsic material robustness and environmentally friendly procedures. The one-pot method used in this work to prepare AuNPs@GA-nanoMIPs meets the requirements for industrial manufacturing processes, and this study is the first report on the current AuNPs@GA-nanoMIP preparation process and its use in GA sensing.

## 4. Conclusions

For the first time, a nanoMIP sensor capable of selectively detecting GA is presented. The novel hybrid polymeric receptor, in situ AuNP-capped AuNPs@GA-nanoMIP, was characterised by FTIR and LSPR, whereas the sensor redox activity due to the presence of the capped AuNPs in the polymer backbones was studied by DPV, EIS and CV. The integration of AuNPs@GA-nanoMIP composites onto the modified SPPtE resulted in a highly sensitive, reproducible GA sensor. Furthermore, this work shows that the Langmuir–Freundlich isothermal (L-FI) model is suitable for characterising the kinetic and binding properties of molecularly imprinted polymers (MIPs), and it allows one to efficiently obtain both parameters regarding the heterogeneity and the saturation of the binding sites, making it a balanced model for describing the adsorption behaviour of MIPs. Finally, this research opens up the way to a valuable alternative to a portable diagnostic platform for the rapid quantification of GA, providing promising perspectives for future on-site applications.

## Figures and Tables

**Table 1:** Adsorption isotherm models adopted in this work.

Isotherm Model	Equation	
Langmuir	$q_{e}=\frac{q_{m}K_{L}\;C_{eq}}{1+K_{L}\;C_{eq}}$	(1)
Freundlich	$q_{e}=K_{F}\;C_{e}^{1/n}$	(2)
Langmuir–Freundlich	$q=\frac{Q_{sat}K\;C^{n}}{1+K\;C^{n}}$	(3)

**Table 2:** Parameters obtained by different isotherm models. To obtain accurate data, the calibration curve was fitted to reach an acceptable R^2^.

	LI			FI			L-FI			
	*Q_max_* (mg/g)	*K_L_* (L/mg)	R^2^	*K_F_* (mg/g)∗(L/mg)^1/n^	*n*	R^2^	*Q_m_* (mg/g)	*K_d_* (M)	*n*	R^2^
AuNPs@GA-nanoMIP	44.18 ± 4.33	1.03 ± 0.25	0.956	19.48 ± 0.35	0.50 ± 0.01	0.994	139.18 ± 36.65	0.05 ± 0.03	0.58 ± 0.02	0.999
AuNPs@nanoNIP	12.35 ± 8.72	0.06 ± 0.04	0.956	0.65 ± 0.03	0.91 ± 0.06	0.961	2.69 ± 0.73	0.43 ± 0.34	0.69 ± 0.08	0.999

**Table 3:** Determination of D-gluconate in spiked red Italian wine using nanoMIP sensor.

	[GA] Added (mg/mL)	[GA] Found (mg/mL)	Recovery (%)
Red Italian wine	0.25	0.235	93.8
	0.50	0.462	92.5
	5.00	5.110	102.2

**Table 4:** A comparison of sensors based on different techniques for the detection of GA.

Receptor	Transduction Material	Method of Detection	Linear Range (mg/mL)	LOD (mg/mL)	RSD (%)	Sample	Reference
Enzymatic	SPCE *	Chronoamperometric	1.6–15.6	1.6	2.9	Spanish red wine	[1]
Enzymatic	SPCE *	Chronoamperometric	1.9–28.7	1.9	8.1	Spanish red wine	[21]
Enzymatic	SPCE *	Chronoamperometric	0.9–135.2	2.6	4.2	Wine and must	[65]
Imprinted nanoparticles	SPPtE **	Voltammetric	0.025–2.5	0.023	6.3	Italian red wine	This work

* Screen-printed carbon electrode; ** screen-printed platinum electrode.

## Data Availability

Data will be provided upon request from the corresponding author.

## Supplementary Materials

The following supporting information can be downloaded at https://www.mdpi.com/article/10.3390/nano15030211/s1, Figures S1–S3—“Molecular modelling”; Figure S4—“DLS measurements”; Figure S5—“EIS investigation”; Figure S6—“DPV responses of AuNPs@nanoNIP (control)”, Figure S7 “Two linear ranges in the calibration curve of the sensor”; Figure S8 “Linearised form of Freundlich isotherm”.
