# Supplementary material for: Adsorption Isotherm Analysis for Hybrid Molecularly Imprinted Polymeric Gold-Decorated Nanoparticles Suitable for Reliable Quantification of Gluconic Acid in Wine
Source: Nanomaterials (Basel). 2025 Jan 28;15(3):211. doi: 10.3390/nano15030211 (PMC11819896; doi:10.3390/nano15030211)
Supplement: Supplementary file 1 [file nanomaterials-15-00211-s001.zip › nanomaterials-3423935-supplementary.pdf]

Supplementary Materials

Adsorption isotherm analysis for hybrid molecular imprinted polymeric gold decorated nanoparticles suitable for reliable quantification of gluconic acid in wine

Nelson Arturo Manrique Rodriguez [1], Marco Costa [1]*, Sabrina Di Masi [1]*, Christopher Zaleski [2], Alvaro García-Cruz [2], Giuseppe Mele[3], Vito Michele Paradiso [4], Sergey Piletsky [2], Cosimino Malitesta [1], Giuseppe Egidio De Benedetto[5]

[1] Laboratory of Analytical Chemistry, Department of Biological and Environmental Sciences and Technologies, University of Salento, Via per Monteroni, 73100 Lecce, Italy; nelsonarturo.manriquerodriguez@studenti.unisalento.it (N.M.-R.); cosimino.malitesta@unisalento.it (C.M.)

[2] Department of Chemistry, University of Leicester, University Rd., Leicester LE1 7RH, UK; cz155@leicester.ac.uk (C.Z.); agc14@leicester.ac.uk (A.G.-C.); sp523@leicester.ac.uk (S.P.)

[3] Department of Engineering of Innovation, University of Salento, via per Arnesano km 1, Lecce 73100, Italy; giuseppe.mele@unisalento.it

[4] Laboratorio di Microbiologia Agraria e Tecnologie Alimentari, Dipartimento di Scienze e Tecnologie Biologiche ed Ambientali, Università del Salento, Via per Monteroni, 73100 Lecce, Italy; vito.paradiso@unisalento.it

[5] Laboratory of Analytical and Isotopic Mass Spectrometry, Department of Cultural Heritage, University of Salento, 73100 Lecce, Italy; giuseppe.debenedetto@unisalento.it

*Correspondence: marco.costa@unisalento.it; sabrina.dimasi@unisalento.it

Contents

S1. Molecular modelling.

S2. DLS measurements.

S3. EIS investigation

S4. DPV responses of AuNPs@nanoNIP (control).

S5. Calibration curve of the sensor

S6. Linearized form of Freundlich isotherm

S7. Interferent responses at 0.025 mg mL-1

S1. Molecular modelling

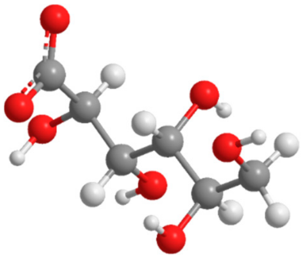

D-gluconate

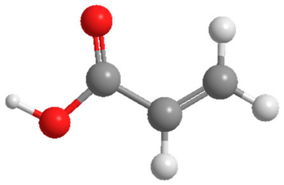

Acrylic acid AA

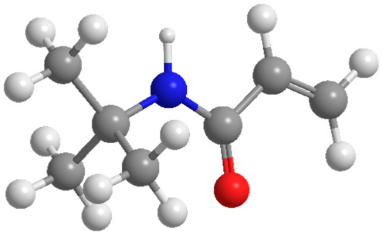

N-Ter-butylacrylamide TBAm

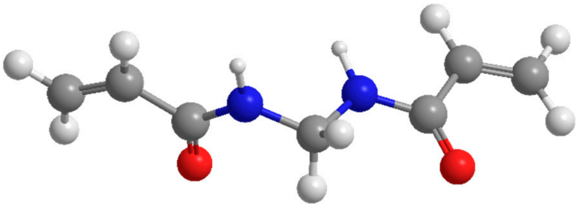

N,N'-Methylenebisacrylamide BIS-MBA

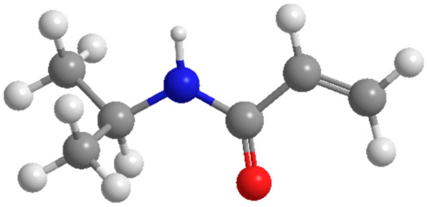

N-Isopropylacrylamide NIPAM.

Figure S1. Optimized structures after MMFF94 minimization in aqueous phase to a minimum energy of 0.001 kcal mol^{-1}.

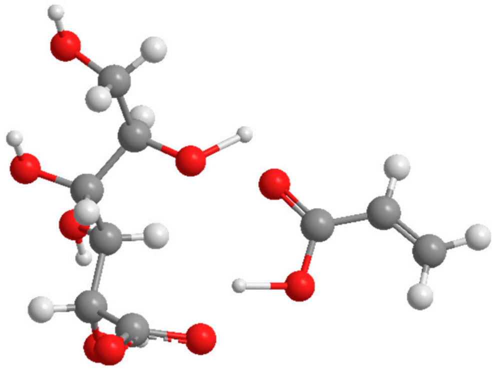

Acrylic acid AA

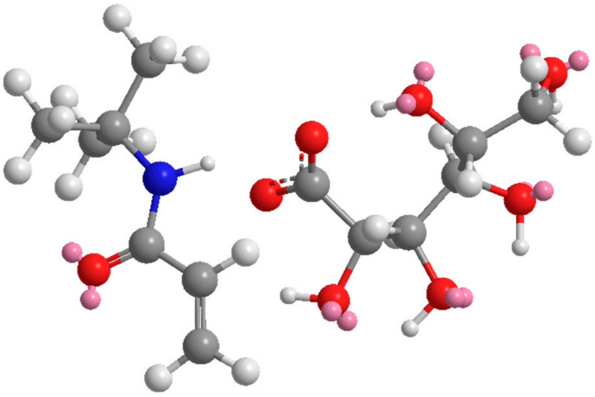

N-Ter-butylacrylamide TBAm

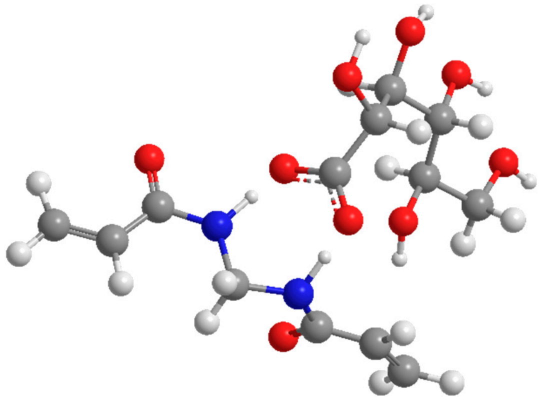

N,N'-Methylenebisacrylamide BIS-MBA

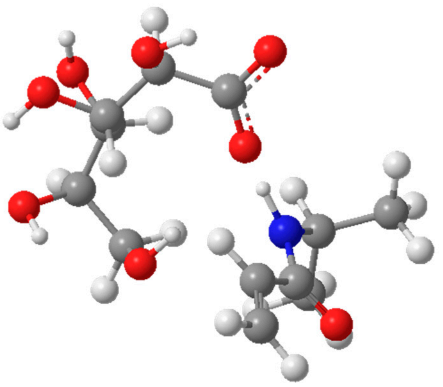

N-Isopropylacrylamide NIPAM

Figure S2. Monomer complex structures and binding scores formed with D-gluconate in aqueous phase for AAc (-16.125 kcal/mol), TBAm (-10.794 kcal/mol), BIS-MBA (-25.472 kcal/mol) and NIPAM (-3.733 kcal/mol).

A

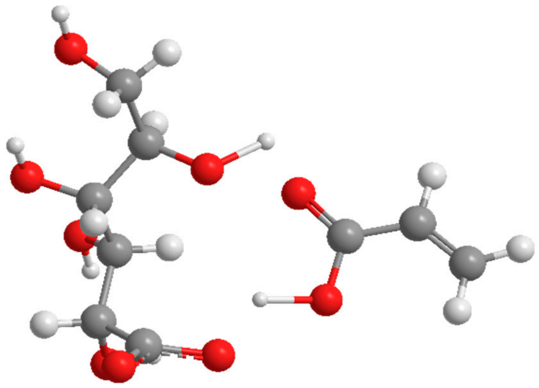

B

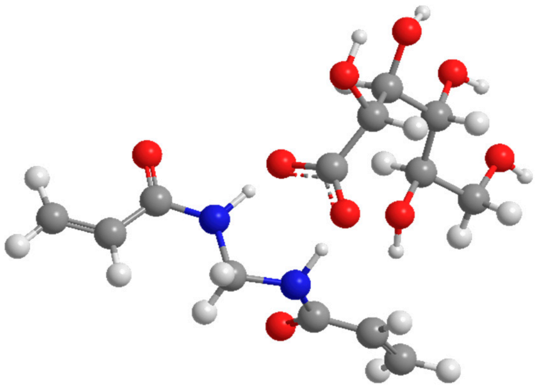

Figure S3. Interaction of monomer interaction with D-gluconate, perfomed by molecular modelling, for: (A) NanoMIP D-gluconate-AA (-16.125 kcal/mol^{-1}) and (B) NanoMIP D-gluconate-MBA (-25.472 kcal/mol^{-1}).

S2. DLS measurements

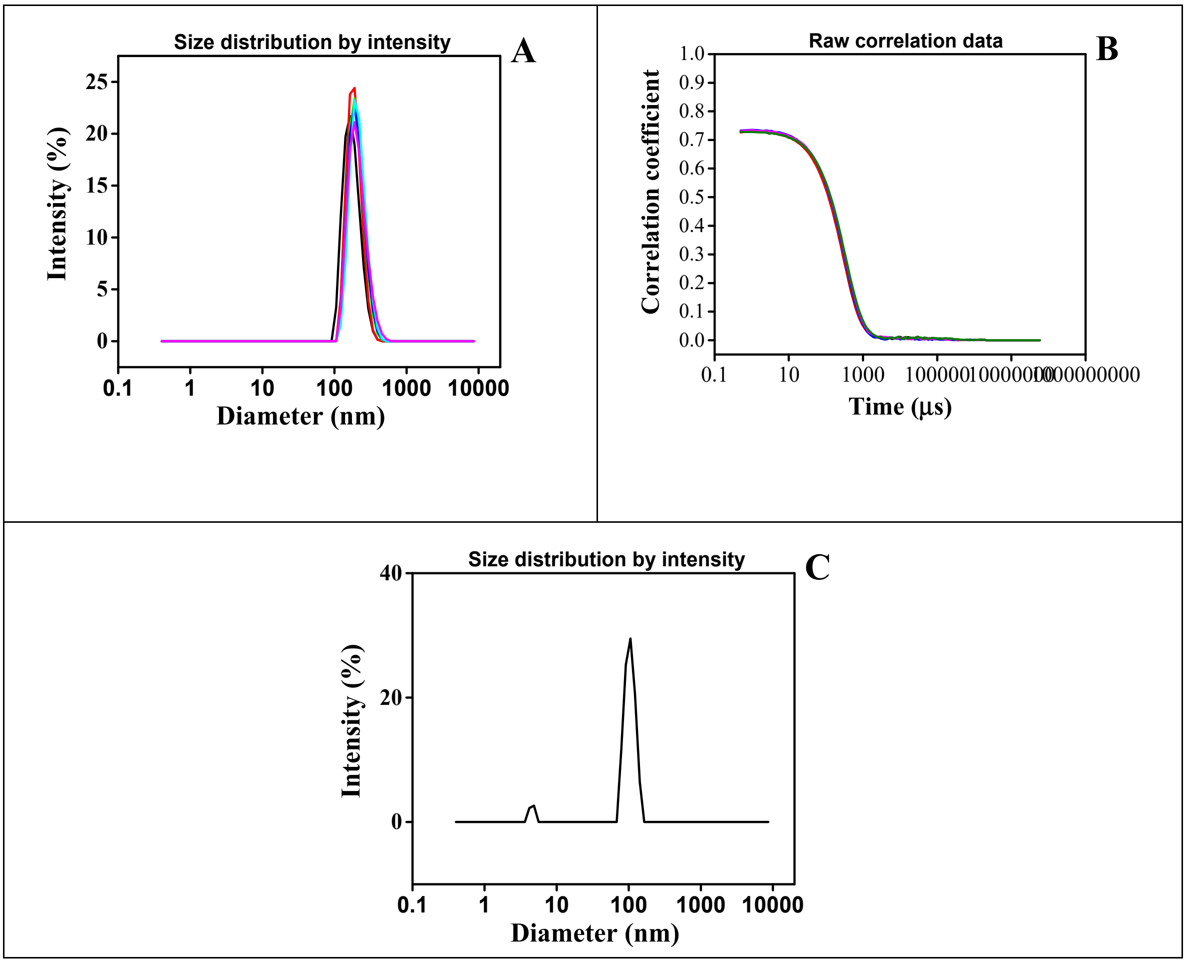

Figure S4. DLS measurement, intensity signal versus particle size (A) and raw correlation data (B). (C) Size distribution of AuNPs.

S3. EIS investigation

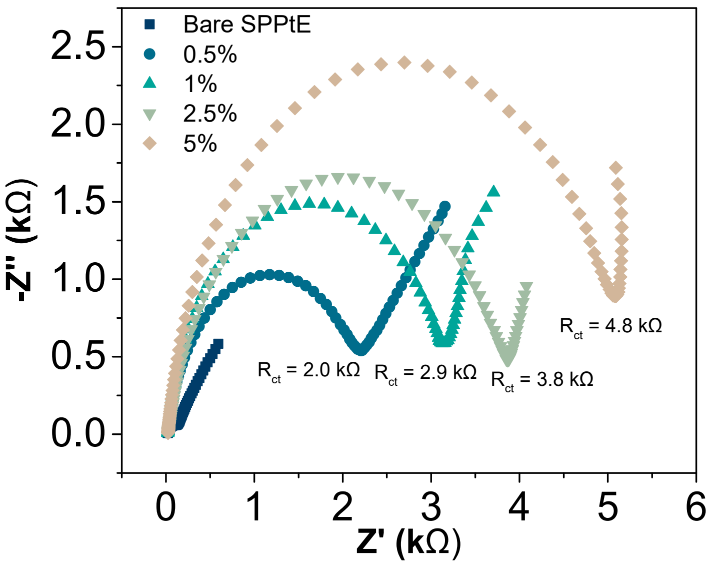

Figure S5. EIS obtained of screen printed platinum electrodes functionalized with different concentrations of APTES (0.5%, 1.0%, 2.5% and 5%).

S4. DPV responses of AuNPs@nanoNIP (control)

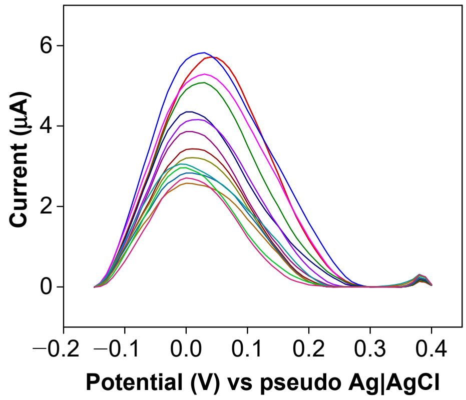

Figure S6. DPV response of the nanoNIP sensor (control) for D-gluconate solutions (concentrations from 0.025 to 5 mg mL^{-1}) in PBS buffer (50 mM, pH= 7.4).

S5. Two linear ranges in calibration curve of the sensor

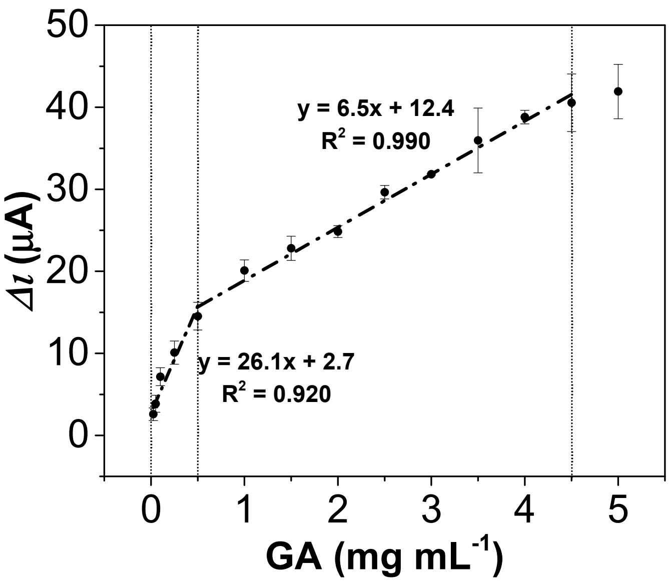

Fig. S7 Calibration curve with two linear range (0 – 0.5 mg/mL, 0.5 – 4.5 mg/mL)

S6. Linearized form of Freundlich isotherm

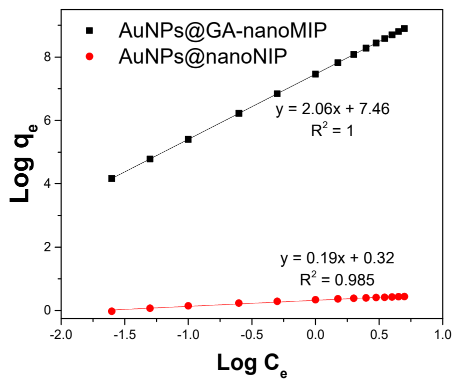

Figure S8. Linearized form of Freundlich isotherm.
